# HOME VIDEO TELEHEALTH TO SUPPORT RURAL VETERAN CAREGIVERS

**DOI:** 10.1093/geroni/igz038.2266

**Published:** 2019-11-08

**Authors:** Bret Hicken, Marren Grant, Christopher Turner, Christy Reynolds, Pamela Wright

**Affiliations:** 1 Veterans Rural Health Resource Center-SLC, Salt Lake City, Utah, United States; 2 VA Caregiver Support Line, Canandaigua, New York, United States

## Abstract

VA offers multiple programs and services to support caregivers of US military Veterans. However, access for rural Veterans and caregivers is challenging due to distance from VA medical facilities. VA Video Connect (VVC) is a remote healthcare platform that enables Veterans to connect directly with VA clinicians through a secure, encrypted video connection. Rural caregivers and Veterans can participate in caregiver services through their own computer or another device from their home, reducing the need for travel to distant VA facilities. In 2018, VA’s Caregiver Support Program and Office of Rural Health developed an implementation pilot to engage eighteen VA Caregiver Support Coordinators (CSCs) in using VVC to monitor and support 180 Veteran/caregiver dyads enrolled in VA’s Program of Comprehensive Assistance for Family Caregivers. The presentation will provide an overview of each phase of implementation, report usability and outcome data from CSCs and caregivers, and discuss implications for broader implementation.

